# Resting Metabolic Rate Does Not Change in Response to Different Types of Training in Subjects with Type 2 Diabetes

**DOI:** 10.3389/fendo.2017.00132

**Published:** 2017-06-13

**Authors:** Kristian Karstoft, Cecilie Fau Brinkløv, Ida Kær Thorsen, Jens Steen Nielsen, Mathias Ried-Larsen

**Affiliations:** ^1^Centre of Inflammation and Metabolism, Rigshospitalet, University of Copenhagen, Copenhagen, Denmark; ^2^Centre for Physical Activity Research, Rigshospitalet, University of Copenhagen, Copenhagen, Denmark; ^3^Department of Endocrinology, Odense University Hospital, Odense, Denmark; ^4^OPEN, Odense Patient Data Explorative Network, Odense University Hospital, Odense, Denmark

**Keywords:** resting metabolic rate, exercise interventions, exercise training, body composition, physical fitness, glycemic control, diabetes type 2

## Abstract

**Background and objectives:**

Ambiguous results have been reported regarding the effects of training on resting metabolic rate (RMR), and the importance of training type and intensity is unclear. Moreover, studies in subjects with type 2 diabetes (T2D) are sparse. In this study, we evaluated the effects of interval and continuous training on RMR in subjects with T2D. Furthermore, we explored the determinants for training-induced alterations in RMR.

**Methods:**

Data from two studies, both including T2D subjects, were encompassed in this manuscript. Study 1 was a randomized, crossover study where subjects (*n* = 14) completed three, 2-week interventions [control, continuous walking training (CWT), interval-walking training (IWT)] separated by washout periods. Training included 10 supervised treadmill sessions, 60 min/session. CWT was performed at moderate walking speed [aiming for 73% of walking peak oxygen uptake (VO_2_peak)], while IWT was performed as alternating 3-min repetitions at slow (54% VO_2_peak) and fast (89% VO_2_peak) walking speed. Study 2 was a single-arm training intervention study where subjects (*n* = 23) were prescribed 12 weeks of free-living IWT (at least 3 sessions/week, 30 min/session). Before and after interventions, RMR, physical fitness, body composition, and glycemic control parameters were assessed.

**Results:**

No overall intervention-induced changes in RMR were seen across the studies, but considerable inter-individual differences in RMR changes were seen in Study 2. At baseline, total body mass (TBM), fat-free mass (FFM), and fat mass were all associated with RMR. Changes in RMR were associated with changes in TBM and fat mass, and subjects who decreased body mass and fat mass also decreased their RMR. No associations were seen between changes in physical fitness, glycemic control, or FFM and changes in RMR.

**Conclusion:**

Neither short-term continuous or interval-type training, nor longer term interval training affects RMR in subjects with T2D when no overall changes in body composition are seen. If training occurs concomitant with a reduction in fat mass, however, RMR is decreased.

**Clinical Trials Registration (www.ClinicalTrials.gov):**

NCT02320526 and NCT02089477.

## Introduction

Most subjects with type 2 diabetes (T2D) are overweight or obese, and overweight/obesity is considered to be a central component of the pathogenesis and pathology of T2D (1, 2). Indeed, weight loss is associated with improvements in glycemic control and other cardiovascular risk factors, and weight loss is recommended for all overweight/obese subjects with T2D (3). Classically spoken, body weight is dependent on the balance between energy intake and energy consumption, and a decrease in energy intake and/or an increase in energy consumption will lead to a weight loss. Energy consumption is dependent on several factors, with resting metabolic rate (RMR) being responsible for 60–70% of the total energy consumption in subjects who are not very active (4). As such, an increase in RMR will increase the likelihood of a weight loss, and interventions that may increase RMR would be attractive in subjects with T2D.

Exercise increases energy expenditure during and after the exercise (5). The increased energy expenditure in the hours following an exercise session is known as excess post-exercise oxygen consumption (EPOC), and this is dependent on both exercise duration and exercise intensity (6). Moreover, training interventions may indirectly increase RMR since fat-free mass (FFM), which is known to be the predominant determinant of RMR (4), is often maintained or increased with training. Since subjects with similar FFM may differ substantially in RMR (7), FFM is, however, not the only determinant of RMR, and although data are conflicting (8), it has been suggested that training may directly influence RMR. As such, it has been found that endurance-trained subjects have higher RMR than sedentary matched controls (9–11) and that training interventions may increase RMR (10, 12). Conversely, other studies have found that training interventions do not affect RMR (13, 14). Whereas these discrepancies between studies may be dependent on different factors, it has been suggested that VO_2_max is an important determinant for changes in RMR (9, 15), and so the ability of a training intervention to increase VO_2_max may be essential.

Only a few studies examining the effect of training interventions on RMR in diabetic subjects have been performed, and, as for healthy subjects, findings are conflicting. Araiza et al. found that a training intervention increased RMR (16), whereas Mourier et al. and Jennings et al. found no effect of training interventions on RMR (17, 18). In subjects with T2D, RMR is typically higher compared to matched normal glucose tolerant subjects, something which is considered to be due to the compromised glycemic control (19, 20). Whereas Araiza et al. found no improvements in glycemic control with their training intervention, both Mourier and Jennings et al. did see training-induced improvements in glycemic control. Thus, it might be speculated whether training-induced improvements in RMR were blunted or even completely offset by the training-induced improvements in glycemic control in the two latter studies.

Exercise intensity is an important determinant for training-induced changes in body composition (21), and we have previously found that 17 weeks of interval-walking training (IWT) results in a substantial weight loss (on average 4 kg) in opposition to time duration and energy-expenditure matched continuous walking training (CWT) (22). Whereas part of this differential weight loss between CWT and IWT may be explained *via* differential EPOC (23), the main reason for the discrepancy remains unclear. There are some indications that training with higher intensity may increase RMR more than training with lower intensity, but it is unclear if this is due to differential effects on VO_2_max and other potential determinants for RMR, or if there is a direct effect of higher training intensity on RMR (24). As such, we aimed to examine the direct effects (independent of changes in body composition and VO_2_max) of short-term (2 weeks) IWT/CWT and the effects of longer term (12 weeks) IWT on RMR in subjects with T2D. Moreover, we aimed to assess the associations between potential determinants for RMR (VO_2_max, body composition, glycemic control) and RMR, both at baseline and in relation to the changes induced with 12 weeks of IWT.

## Materials and Methods

This manuscript builds on data from two different studies, both including subjects with T2D (25). Exclusion criteria were pregnancy, smoking, contraindication to increased levels of physical activity (26), insulin dependence, and evidence of thyroid, liver, lung, heart, or kidney disease. All subjects underwent a screening consisting of a medical interview and examination, an oral glucose tolerance test (OGTT), a walking VO_2_peak test with indirect calorimetry (Cosmed K4B2, Rome, Italy) and a familiarization VO_2_max test performed on a treadmill (Katana Sport, Lode, Groningen, the Netherlands) with indirect calorimetry (Cosmed Quark, Rome, Italy) as previously described (22, 27). Written and informed consent was obtained from all research participants before any investigations were performed and the studies were approved by the Ethical Committee of the Capital Region of Denmark (H-6-2014-043 and H-1-2013-116) and registered at www.ClinicalTrials.gov (NCT02320526) and (NCT02089477).

### Study Designs and Interventions

Study 1 was a randomized, crossover trial where subjects were included in three different interventions, each lasting 2 weeks. The interventions were CWT (ten 60-min walking sessions performed with a continuous speed, aiming for oxygen uptake rates at 73% of VO_2_peak); IWT [ten 60-min walking sessions performed with cycles of alternating 3-min slow (54% of VO_2_peak) and 3-min fast (89% of VO_2_peak) walking]; control (CON) (no walking), and interventions were performed in randomized order. All walking sessions were performed at a treadmill (Katana Sport) and controlled with indirect calorimetry at the first and sixth session (in order to determinate the walking speed that corresponded to the correct oxygen uptake rates). Between interventions, washout periods (8 weeks after CWT/IWT, 4 weeks after CON), where subjects returned to their habitual activity level, were applied to ensure that any intervention-induced effects disappeared before initiation of the next intervention. Other data from this study have previously been published (28).

Study 2 was a single-arm intervention study, where subjects were prescribed free-living IWT for 12 weeks. Subjects were told to complete at least three weekly training sessions, each lasting at least 30 min and with repeated cycles of 3-min fast and 3-min slow walking. Training was controlled by a smartphone application (InterWalk^®^), and data from training sessions were uploaded to a central server (29). Other data from this study have previously been published (27).

### Investigations

Before (pre) and after (post) interventions, subjects underwent one experimental day (meaning that subjects in Study 1 completed 6 experimental days in total). Subjects met fasting (~12 h for all except water) in the laboratory, by means of passive transport (car, bus, etc.). After confirming that no subjective feeling of acute disease and no fever was present, subjects voided. Subjects were then weighted, had an antecubital vein catheter inserted, and were placed in a bed in a temperature controlled (20°C) and calm room. After an acclimatization period of at least 30 min, the RMR measurements commenced: a standardized head tilt (15°) was applied to the bed and a ventilated hood (Cosmed, rounded canopy) was placed over the subject’s head and connected to an indirect calorimetric system (Cosmed Quark) *via* a canopy blower (Cosmed). Carbon dioxide concentrations in the system were kept below 1% to avoid excess breathing (30). Subjects were instructed to breathe normally and not to fall asleep. RMR measurements were performed for 20 min.

Following the RMR measurements, fasting blood samples (lithium-heparin and EDTA tubes) were obtained and subjects included in Study 1 underwent supine resting whereas subjects in Study 2 underwent a 2 h standard OGTT (75 g anhydrate glucose dissolved in water to a total volume of 300 ml) with bedside blood glucose measurements (ABL 8 series, Radiometer, Herlev, Denmark) obtained every 30 min. Finally, following resting/OGTT procedures, all subjects were given a light meal and underwent a dual-energy X-ray absorptiometry scan (Lunar Prodigy Advance; GE Healthcare, Madison, WI, USA) and a VO_2_max test comparable to the one performed at the screening day.

Post-intervention investigations were in Study 1 initiated 39–43 h after the last exercise bout (in CWT/IWT interventions), and in Study 2 at least 48 h after the last exercise bout.

### Analyses and Calculations

Fasting blood samples were centrifuged (2,000 g, 15 min, 4°C). Lithium-heparin plasma was analyzed for thyroid hormones (thyroid-stimulating hormone, triiodothyronine, and thyroxine) and insulin *via* Electrochemiluminescence immunoassay (Cobas 8000, Roche Diagnostics, IN, USA). EDTA plasma was analyzed for HbA1c *via* absorption photometry (Tosoh G7; Tosoh, San Francisco, CA, USA).

Mean oxygen uptake and carbohydrate excretion rates were calculated from the indirect calorimetric measurements. RMR was calculated according to the equations by Weir (31).

### Statistics

First, intervention-induced effects on RMR were compared using two-way (time × intervention) repeated-measures (RM) ANOVA (Study 1) and Student’s paired *t*-test (Study 2).

Next, simple linear regression analyses between potential determinants of RMR (VO_2_max, body composition, and glycemic control variables) and RMR were performed on baseline data (both studies) and on post–pre intervention (delta) values (Study 2). To avoid regression toward the mean, all delta values were controlled for baseline values, and this did not change the results of the regression analyses.

Finally, due to large between-subject heterogeneity in RMR responses in Study 2, subjects were stratified into three groups according to the intervention-induced effect on RMR as (1) decreased RMR (≥ 10% decrease); (2) unchanged RMR; (3) increased RMR (≥10% increase). The specific cutoff levels were chosen to ensure that subjects categorized in group 1 and 3 with certainty had intervention-induced alterations in RMR and that the differences measured were not just due to imprecision of the measurements or biological day-to-day variation (30, 32). Stratified analyses were performed as one-way ANOVA of baseline values (to assess baseline differences between strata), as one-way RM ANOVA of delta values between strata (to assess differential changes in potential determinants of RMR between strata), and as two-way (time × stratification) RM ANOVA’s (to assess differential changes in potential determinants of RMR within each strata).

Data are reported as mean ± SEM or delta values with confidence intervals (CI). All analyses were performed using Prism v6.03 (Graphpad Software, CA, USA) and statistical significance was accepted when *p* < 0.05.

## Results

Baseline data are given in Table 1. *N* = 14 subjects were included in Study 1 with all subjects being included in the analyses. *N* = 32 subjects were included in Study 2, but only 23 subjects underwent RMR measurements. As such, *N* = 37 subjects were overall included in the current analyses. No subjects changed glucose-lowering medication during the study period. In Study 1, glucose-lowering medication was continued unchanged during the entire study, whereas, in Study 2, glucose-lowering medication was paused from 2 days before each experimental day and until the end of the experimental day.

**Table 1:** Pre- and (for Study 2) post-intervention characteristics.

	Study 1	Study 2 pre	Study 2 post
*n*	14	23	
Sex (M/F)	11/3	7/16	
Age (years)	65.3 ± 1.7	64.8 ± 1.5	
Time since diagnosis (years)	8.6 ± 1.3	6.0 ± 0.9	

**Glucose-lowering medication (*n*)**			
Metformin	14	19	
Sulfonylureas	3	2	
GLP-1 analogs/DPP-4 inhibitors	3	7	
SGLT2 inhibitors	0	1	

**RMR**			
Absolute (ml O_2_/min)	1,736 ± 85	1,659 ± 51	1,646 ± 69
Relative to body mass (ml O_2_/min/kg TBM)	18.1 ± 0.6	21.4 ± 0.8*	21.0 ± 0.7
Relative to FFM (ml O_2_/min/kg FFM)	28.8 ± 1.1	34.4 ± 0.8*	34.0 ± 1.1

**Physical fitness (VO_**2**_max)**			
Absolute (ml O_2_/min)	2,438 ± 147	1,961 ± 90	2,065 ± 92^‡^
Relative to body mass (ml O_2_/min/kg TBM)	25.3 ± 1.1	25.1 ± 1.0	26.3 ± 0.8^‡^
Relative to FFM (ml O_2_/min/kg FFM)	41.3 ± 3.7	40.0 ± 1.1	42.1 ± 0.8^(‡)^

**Body composition**			
BMI (kg/m^2^)	31.6 ± 1.1	28.8 ± 1.3	28.7 ± 1.2
TBM (kg)	98.3 ± 4.7	79.7 ± 3.5*	79.3 ± 3.4
FFM (kg)	61.5 ± 3.2	48.8 ± 1.8*	48.7 ± 1.8
Fat mass (kg)	36.8 ± 2.1	30.6 ± 2.5	30.2 ± 2.5
Fat percentage (%)	38.0 ± 1.5	38.8 ± 2.0	38.5 ± 2.1

**Glycemic control**			
Fasting glucose (mmol/l)	7.7 ± 0.5	6.9 ± 0.4	7.1 ± 0.4
Fasting insulin (pmol/l)	119 ± 35	72 ± 10	83 ± 10
Two-hour OGTT glucose (mmol/l)	13.1 ± 1.3	14.4 ± 0.7	14.1 ± 0.7
Mean OGTT glucose	13.0 ± 0.7	13.4 ± 0.6	13.2 ± 0.6
HbA1c (mmol/mol)	47.7 ± 2.4	50.1 ± 2.5	50.5 ± 2.3

**Thyroid hormones**			
TSH (×10^−3^ IU/L)	1.8 ± 0.3	1.6 ± 0.2	1.7 ± 0.2
Triiodothyronine (nmol/l)	1.6 ± 0.1	1.6 ± 0.1	1.6 ± 0.1
Thyroxine (nmol/l)	96.7 ± 6.1	87.3 ± 2.7	89.5 ± 2.9

*Data are numbers (*n*) or mean ± SEM*.

*GLP-1, glucagon-like peptide-1; DPP-4, dipeptidyl peptidase-4; SGLT2, sodium-glucose cotransporter-2; RMR, resting metabolic rate; VO_2_max, maximal oxygen consumption rate; TBM, total body mass; FFM, fat-free mass; BMI, body mass index; OGTT, oral glucose tolerance test; HbA1c, hemoglobin A1c; TSH, thyroid-stimulating hormone*.

Statistical differences are indicated by *(*p* < 0.05, Study 1 vs. Study 2 pre, Student’s unpaired *t*-test) and ^‡^(*p* < 0.05, Study 2 pre vs. study 2 post, Student’s paired *t*-test). ^(‡)^indicates *p* < 0.10.

### Training Data

In Study 1, training adherence (amount of training performed relative to prescribed) was 99% in both CWT and IWT. As previously published (28), mean oxygen consumption and heart rates were comparable between CWT and IWT, whereas fast and slow IWT intervals were performed with higher and lower oxygen consumption and heart rates, respectively, compared to CWT.

In Study 2, the mean uploaded IWT time was 68 ± 9 min/week, corresponding to 75% of the minimal volumes of prescribed training. There were, however, substantial between-subject differences in uploaded IWT time, with six individuals uploading less than 30% of the minimal volumes of prescribed training. If excluding these six, apparently non-adherent, subjects from the analyses, mean uploaded IWT time was 85 ± 7 min/week, corresponding to 94% of the minimal amounts of prescribed training. It was not possible to assess training intensity from the uploaded data.

### Intervention-Induced Effects on RMR

**Figure 1:**
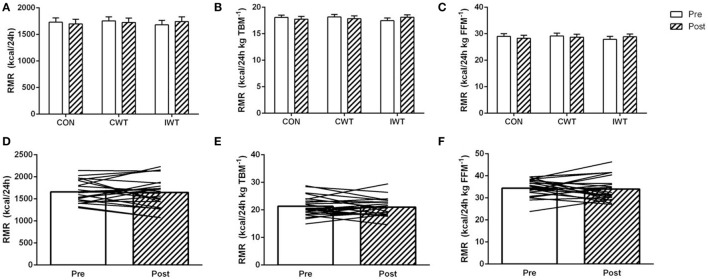
The effect of training interventions on resting metabolic rate (RMR). Subjects with type 2 diabetes underwent three 2-week interventions **(A–C)**; no training [control (CON)], continuous walking training (CWT), interval-walking training [IWT or 12 weeks of IWT training **(D–F)**]. RMR was measured before and after interventions and is reported as total RMR **(A,D)**, RMR relative to total body mass [TBM **(B,E)**] and RMR relative to fat-free mass [FFM **(C,F)**]. Data are shown as mean ± SEM **(A–C)** and mean + individual data **(D–F)**. Statistical analyses [two-way repeated-measures ANOVA in Panels **(A–C)**] and Student’s paired *t*-test **(D–F)** did not result in any significant changes within interventions (*p* > 0.05 for all comparisons).

In Study 1, no effect of any intervention was found on RMR [delta CON = −33 (95% CI: −122 to 57) kcal/24 h, delta CWT = −32 (95% CI: −122 to 58) kcal/24 h, delta IWT = 62 (95% CI: −28 to 152) kcal/24 h, *p* > 0.05 for all comparisons] (Figure 1). Likewise, in Study 2, no overall intervention-induced change in RMR was found [delta IWT = −13 (95% CI: −125 to 98) kcal/24 h, *p* > 0.05], and exclusion of the subjects who were apparently non-adherent to the training (*n* = 6), did not change this. Moreover, no association was seen between uploaded IWT time and changes in RMR (*r*^2^ = 0.05, *p* = 0.34) However, intervention-induced changes in RMR varied considerably between subjects (Figures 1D–F). As such, *n* = 7 subjects decreased RMR (≥10%), *n* = 9 subjects did not change RMR and *n* = 7 subjects increased RMR (≥10%) with the intervention. Normalization of RMR to total body mass (TBM) or FFM did not alter the above-standing results.

### Potential Determinants of RMR

In Study 1, no intervention-induced effects on physical fitness or body composition was seen with any of the interventions (*p* > 0.05 for all comparisons, data not shown) (Table 1). Conversely, and as previously described (28), measures of glycemic control (mean and maximum 24 h glucose levels) were improved with IWT, with no effects of CON or CWT.

In Study 2, physical fitness improved with the intervention [delta VO_2_max = 104 (CI: 11–197) ml/min, *p* < 0.05], whereas neither body compositional nor glycemic control variables improved with the intervention (Table 1, *p* > 0.05 for all comparisons).

### Associations between Potential RMR-Determinants and RMR

**Figure 2:**
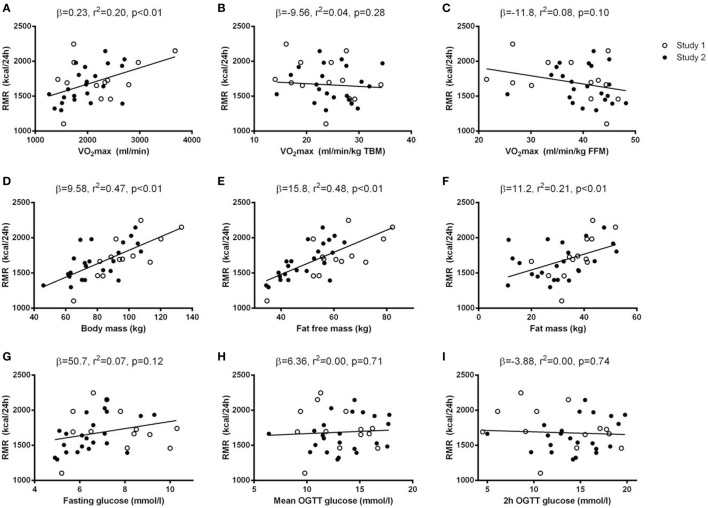
Baseline associations between resting metabolic rate (RMR) and potential determinants of RMR. Simple regression analyses were performed between baseline values of potential determinants of RMR (*x*-axis) and baseline RMR (*y*-axis). The potential determinants were VO_2_max [absolute, relative to total body mass (TBM), and relative to fat-free mass (FFM) **(A–C)**], body composition [body mass, FFM, and fat mass **(D–F)**] and glycemic control [fasting glucose, mean oral glucose tolerance test (OGTT) glucose, and 2 h OGTT glucose **(G–I)**]. Data from both Study 1 (open circles) and Study 2 (black circles) were included in the regression analyses and results (β-coefficients, *r*^2^-, and *p*-values) are given in each panel.

**Figure 3:**
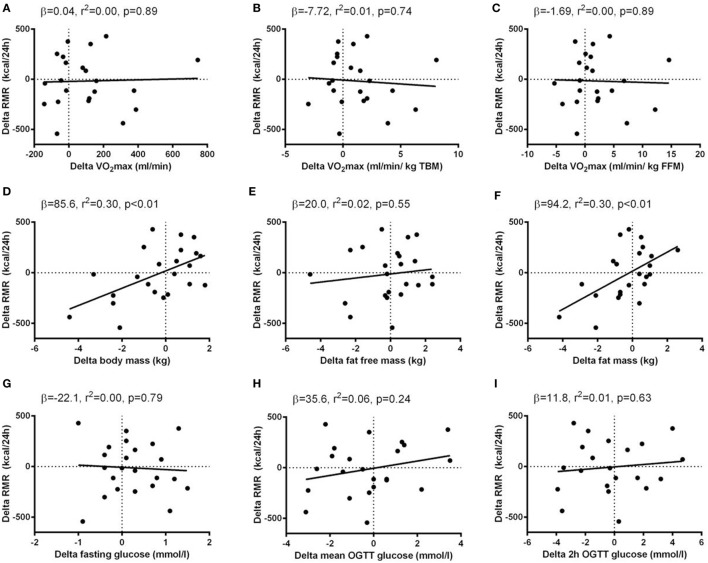
Associations between delta (post minus pre intervention) values of resting metabolic rate (RMR) and delta values of potential determinants of RMR. Simple regression analyses were performed between delta values of potential determinants of RMR (*x*-axis) and delta values of RMR (*y*-axis). The potential determinants were VO_2_max [absolute, relative to total body mass (TBM) and relative to fat-free mass (FFM) **(A–C)**], body composition [body mass, FFM and fat mass **(D–F)**] and glycemic control [fasting glucose, mean oral glucose tolerance test (OGTT) glucose, and 2 h OGTT glucose **(G–I)**]. Data from Study 2 were included in the regression analyses and results (β-coefficients, *r*^2^-, and *p*-values) are given in each panel.

Baseline levels of VO_2_max were positively correlated with RMR (Figures 2 and 3). When normalizing VO_2_max to body mass or FFM, however, the association disappeared. No significant associations between delta values in VO_2_max and RMR were seen.

Body compositional variables (TBM, FFM, and fat mass), were all positively correlated with RMR at baseline. When analyzing delta values, the associations between TBM/fat mass and RMR were maintained, whereas no association between FFM and RMR was seen. The association between changes in fat mass and RMR was maintained when a sequential correction for changes in the other potential RMR-determinants was performed.

No associations between glycemic control variables (fasting glucose, mean OGTT glucose, 2 h OGTT glucose, HbA1c) and RMR were seen, neither at baseline nor when analyzing delta values.

### Changes in Potential RMR-Determinants in Stratified Analyses

**Figure 4:**
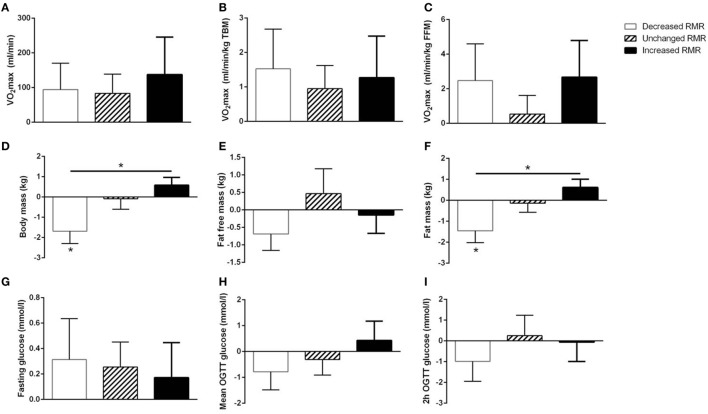
Stratified analyses of potential determinants for resting metabolic rate (RMR). Subjects included in Study 2 were stratified according to their change in RMR as increased RMR (≥10%, *n* = 7), unchanged RMR (*n* = 9), or decreased RMR (≥10%, *n* = 7). Delta (post minus pre intervention) values ± SEM of potential determinants for RMR {VO_2_max [absolute, relative to total body mass (TBM), and relative to fat free mass (FFM); panel **(A–C)**], body composition [body mass, fat free mass, and fat mass; panel **(D–F)**], and glycemic control [fasting glucose, mean OGTT glucose, and 2 h OGTT glucose; panel **(G–I)**]} are shown for the different strata. Within-strata changes in potential determinants of RMR were analyzed by two-way (strata × time) repeated-measures (RM) ANOVA (significant changes indicated by an asterisk above the bar) and between-strata differences were analyzed by one-way RM ANOVA of the delta values (significant changes indicated by a connecting line and an asterisk).

No baseline differences in RMR or any potential determinants of RMR (measures of VO_2_max, body compositional variables, glycemic control variables) were seen between strata (*p* > 0.05 for all comparisons) (Table 2; Figure 4).

**Table 2:** Stratified analyses in Study 2.

	Decreased RMR		Unchanged RMR		Increased RMR	
	Pre	Post	Pre	Post	Pre	Post
**RMR**						
Total (kcal/24 h)^†#^	1,616 ± 102	1,308 ± 69*	1,751 ± 85	1,735 ± 95	1,586 ± 78	1,871 ± 92*
Relative to body mass (kcal/24 h/kg TBM)^#^	36.4 ± 0.9	30.0 ± 0.6*	34.9 ± 1.2	34.4 ± 1.7	31.8 ± 1.7	37.6 ± 2.1*
Relative to FFM (kcal/24 h/kg FFM)^#^	23.7 ± 1.4	19.7 ± 1.2*	21.6 ± 1.0	21.4 ± 1.0	18.7 ± 1.1	21.9 ± 1.4*

**Physical fitness (VO_2_max)**						
Absolute (ml O_2_/min)^‡^	1,681 ± 88	1,721 ± 154	2,141 ± 135	2,225 ± 151	2,034 ± 190	2,171 ± 146
Relative to body mass (ml O_2_/min/kg TBM)^‡^	24.8 ± 1.6	25.3 ± 1.0	28.2 ± 2.2	29.2 ± 2.3	24.0 ± 2.2	25.2 ± 1.4
Relative to FFM (ml O_2_/min/kg FFM)^(‡)^	38.0 ± 1.0	40.5 ± 2.3	43.3 ± 2.2	43.9 ± 2.3	40.4 ± 3.0	43.1 ± 1.2

**Body composition**						
Body mass (kg)^#^	70.1 ± 7.0	68.4 ± 6.4*	82.0 ± 5.1	81.9 ± 4.8	86.4 ± 5.7	87.0 ± 5.6
FFM (kg)	44.6 ± 3.2	43.9 ± 3.1	50.6 ± 3.0	51.1 ± 3.0	50.6 ± 3.3	50.5 ± 3.1
Fat mass (kg)^#^	25.4 ± 5.0	24.0 ± 4.6*	30.9 ± 4.2	30.7 ± 4.2	35.2 ± 3.4	35.9 ± 3.4
Fat percentage (%)	36.0 ± 3.9	35.2 ± 4.0	38.2 ± 3.8	37.9 ± 3.9	42.2 ± 2.1	42.7 ± 2.0

**Glycemic control**						
Fasting glucose (mmol/l)	6.2 ± 0.5	6.5 ± 0.6	6.5 ± 0.2	6.7 ± 0.2	8.0 ± 1.2	8.2 ± 1.0
2 h OGTT glucose (mmol/l)^(†)^	15.5 ± 0.9	14.5 ± 1.4	12.4 ± 1.4	12.6 ± 1.0	15.8 ± 1.2	15.7 ± 1.1
Mean OGTT glucose (mmol/l)^(†)^	13.8 ± 0.9	13.0 ± 1.3	12.2 ± 0.9	11.9 ± 0.6	14.7 ± 1.1	15.1 ± 0.7
HbA1c (mmol/mol)	49.9 ± 4.6	48.6 ± 3.4	47.1 ± 1.2	48.6 ± 1.6	53.9 ± 6.2	54.7 ± 6.4

*Subjects with type 2 diabetes underwent a 12-week interval-walking training intervention, with measurements of resting metabolic rate (RMR), physical fitness, body composition, and glycemic control before (Pre) and after (Post) the intervention. Subjects were stratified according to the intervention-induced change in RMR as decreased RMR (≥10%), unchanged RMR or increased RMR (≥10%), and the intervention-induced changes in potential determinants of RMR were analyzed using a two-way repeated-measures ANOVA for within-strata changes. Data are presented as mean ± SEM*.

Statistical differences are indicated by ^‡^(main effect of time), ^†^(main effect of stratification), ^#^(time × stratification interaction), *(within group, pre vs. post, *p* < 0.05). Parenthesis indicates *p* < 0.10.

*TBM, total body mass; FFM, fat-free mass; OGTT, oral glucose tolerance test; HbA1c, hemoglobin A1c*.

For measures of VO_2_max and glycemic control, no intervention-induced changes within strata were seen, nor were there any intervention-induced differences between strata.

An intervention-induced reduction in body mass was seen in subjects who also decreased RMR, and a between-strata difference in body mass was seen between subjects who decreased and subjects who increased RMR (*p* < 0.05 for both).

Whereas the results for fat mass mirrored those seen for TBM, no differences within or between strata was seen for FFM.

### Hormone Levels

In Study 1, no intervention-induced effects on fasting insulin or thyroid hormones were seen with any of the interventions (data not shown, *p* > 0.05 for all comparisons).

In Study 2, fasting insulin and thyroid hormones did not change with the intervention, and likewise, no differences were seen in the stratified analyses (data not shown, *p* > 0.05 for all comparisons).

## Discussion

The most important finding of this study is that neither short-term continuous or interval-based training nor longer term interval-based training altered RMR in subjects with T2D as long as the training did not alter body composition. Body composition, both FFM and fat mass, were important determinants for RMR at baseline, but, interestingly, only training-induced changes in fat mass and not in FFM were associated with training-induced changes in RMR. This was supported by the stratified analyses, were subjects with a training-induced loss of fat mass had an accompanying decrease in RMR.

The lack of training-induced changes in RMR is in line with most previous studies. Both in healthy subjects (13, 14) and in subjects with T2D (17, 18), it is most commonly reported that RMR does not change with a training intervention. However, some studies have found increased RMR after a training intervention (10, 12, 16). Whereas parts of the explanation for the conflicting findings may be due to different training modalities (33), and differential changes in body composition, it is also possible that the post-intervention RMR measurement has been performed too early after the last exercise bout in some studies, implying that EPOC has been included in the measurement (6). Whereas we did not see any significant changes in RMR in any of the two studies in the primary analyses, a paired *t*-test indicated a tendency for increased RMR with IWT in Study 1 (*p* = 0.06). Since EPOC is increased with IWT compared to both CON and CWT (23), and since our RMR measurements were performed ~40 h after the last exercise bout, it is possible that the tendency for increased RMR seen with IWT in Study 1 in fact was prolonged EPOC (8).

In contrast to previous observations (19, 20), we did not find any indication that glycemic control affected RMR. Increased RMR has mainly been reported in subjects with dysregulated diabetes (5) and the subjects included in our studies had a fairly good glycemic control both at baseline and after the interventions; potentially too good to affect RMR. Also, the previously reported positive correlation between VO_2_max and RMR (9, 15), was not replicated in our data when VO_2_max relative to body weight or FFM was used, neither for baseline values nor for intervention-induced changes. Since an association between changes in VO_2_max and glycemic control has previously been described in subjects with T2D (34), it would hypothetically be possible that subjects who increased VO_2_max the most also improved glycemic control the most, and that the combined and opposing effect of these determinants resulted in no changes in RMR. However, since no associations were seen between changes in VO_2_max/glycemic control and changes in RMR and since no associations were found between changes in VO_2_max and changes in glycemic control (data not shown), we find this unlikely.

The strong baseline associations we found between FFM and fat mass on one side and RMR on the other side have previously been reported (35). Interestingly, when comparing intervention-induced changes in Study 2, the association between FFM and RMR disappeared, whereas the association between fat mass and RMR persisted. Moreover, subjects who decreased RMR with the training intervention also lost fat mass. Whereas FFM is considered to be the primary determinant for RMR and training-induced changes in RMR are most often explained by changes in FFM (4, 8), it has also been reported that a training-induced loss of fat mass may “overrule” the effect of an increase in FFM on RMR since this combination has been shown to decrease RMR (36). The mechanisms underlying these results cannot readily be derived from our data. It is generally believed, however, that the body responds to a weight loss with a homeostatic energy sparring, which is mainly seen as decreased RMR dependent on reductions in hormones like insulin and triiodothyronine (37) and reduced activity of the sympathetic nervous system (38). This has mainly been shown for a weight loss arising from dietary energy restriction (39–41), but may also be seen when at least parts of the weight loss is mediated *via* increased physical activity (42, 43). Whereas we did not see any changes in insulin or thyroid hormones with any of the training interventions, we did not measure sympathetic nervous system activity. Since changes in sympathetic nervous system activity are closer associated with changes in fat mass than with changes in FFM (44), it is plausible that the subjects who lost fat mass in Study 2 had a reduction in RMR due to a decreased activity of the sympathetic nervous system.

While Study 1 was fully supervised efficacy trial with high training adherence, Study 2 was a free-living effectiveness trial without supervision. Whereas the training adherence in Study 2, in terms of volume, was fairly good compared to other free-living training studies (45, 46), the training adherence, in terms of intensity, was not possible to assess. Since no overall changes in glycemic control and body composition were seen in Study 2, it must be speculated how good the training adherence was. Subjects improved their VO_2_max, however, indicating that some effect of the training was seen. Still, when compared to our previous study, where 17 weeks of IWT resulted in considerable weight loss and improvements in glycemic control and a much greater increase in VO_2_max (22), it is clear that subjects included in the current Study 2 had suboptimal training responses. The prescribed volume of IWT (at least three times a week with at least 30 min duration pr. session) in the current Study 2 was approximately 1/3 of the previously prescribed training volume (22), indicating that at least training volume is important for the metabolic improvements seen. As such, and given that higher training volume and intensity (24) and greater improvements in VO_2_max (9, 15) may be central for increases in RMR, it may be speculated whether a more intense training intervention would have resulted in different RMR results.

Given that the analyses in this paper were secondary to other analyses (27, 28) and that no power calculations were performed for RMR outcomes, it may be speculated whether the negative findings were a result of a lack of power. This is further important since a large heterogeneity was seen for changes in RMR. As such, despite the overall analyses indicated that RMR was unaffected by the training interventions, no final conclusions can be drawn from this paper.

In summary, neither short-term continuous or interval-type training, nor longer term interval training affects RMR in subjects with T2D when no overall changes in body composition are seen. Whereas both FFM and fat mass are important determinants of RMR at baseline, only training-induced changes in fat mass and not in FFM seem to be important for training-induced changes in RMR.

## Author Contributions

KK designed the studies, analyzed and interpreted the data, and wrote the manuscript. MR-L contributed to the study design. KK, CB, IT, and JN researched the data. All authors reviewed and revised the manuscript, approved the final version, and agreed to be accountable for the content of the work.

## Conflict of Interest Statement

The authors declare that the research was conducted in the absence of any commercial or financial relationships that could be construed as a potential conflict of interest. The reviewer, OS, and handling editor declared their shared affiliation, and the handling editor states that the process nevertheless met the standards of a fair and objective review.
